# The Element of Causation in Abdominal Contusions*Read at the annual meeting of the New York State Medical Association, October 12, 1894

**Published:** 1895-02

**Authors:** Thomas H. Manley

**Affiliations:** 115 West Forty-ninth Street; New York


### Southern Medical Record.

A MONTHLY JOURNAL OF MEDICINE AND SURGERY.

VOL. XXV. ATLANTA, GA., FEBRUARY, 1895. No. 2.

### Original Articles.

### THE ELEMENT OF CAUSATION IN ABDOMINAL CONTUSIONS.*

BY THOMAS H. MANLEY, M. D., NEW YORK.

(CONCLUDED.)

The intestine being a suspended, floating body, when sudden and violent force is applied, it is contused or rent, in proportion to the momentum and volume of force sustained. Nevertheless, abdominal viscera seldom sustain serious harm from blows; except when they are directed with great energy; as the kick of a horse; when the parts are crushed, as well as contused; and laceration follows, not through the impetus of impact, but, through the viscera being directly compressed against the spine or pelvic brim.

This is because the abdominal walls offer great resistance and the velocity of impact is not ample to disrupture the mobile, elastic bowel, except in unusual cases.

A young man came under my care a year ago, who illustrated the *modus operandi* of contusive force on the abdomen. He was running a circular saw, when the board in his hand was suddenly splintered, a large fragment hitting him with great force over the umbilical region. He died three days later, when an extensive rupture of the jejunum was discovered.

Another case which typifies the same class, came under my care in July of this year. A large, heavy man was violently

*Read at the annual meeting of the New York State Medical Association, October 12, 1894

kicked by a young colt, over the abdomen. Collapse promptly set in and he died within six hours; probably, from internal hemorrhage.

An autopsy was denied.

A swimmer, as he plunges into the water, does so head first, as he knows from experience, severe shock is likely to be produced by striking flat on the abdomen.

On the whole, concussive trauma of the abdomen is more common than compressive, though, with few exceptions, the consequences are more trivial.

Secondly. *Crushing Injuries* of the abdomen are always the most severe and yield the largest mortality. One is crushed by some falling body, by the wheels of a vehicle passing over the trunk; or by being jammed between two resisting bodies.

The mobile abdominal walls anteriorly retract away from the advancing force, until they are arrested by the vertebral barrier behind.

The organs are caught between the opposing surfaces within, and traumatized, in various degrees. In some, in consequence of moderate force, the extent of contusion is slight, with no marked constitutional disturbances, while with others, when ponderous bodies have passed over the abdomen, or the extent of compression has been great, a solid organ may be reduced to a pulp, terribly lacerated or contused. The hollow organs, as the intestine and canals, suffer variously; from a slight abrasion of the mucous or serous coat, to a complete cleavage through its lumen.

The precise manner in which various types of visceral injury follow the application of violence over the belly's surface, is not by any means clear.

How, indeed, in one case the common bile duct suffers perforation from pressure; when the muscular-girth, through which that force must be transmitted, the overlying, fragile intestine and the adjacent friable liver, all escape, is quite beyond our comprehension or knowledge.

I have elsewhere reported a remarkable case of rupture of the receptaculum chyli—all the other organs (*Medical News*) escaping serious damage. The mesenteric vessels may be opened, the ureter lacerated or nerve-trunks torn in two; and yet the circumjacent viscera escape. And it certainly is a most remarkable phenomena that deeply lodged organs may bear serious mutilation, and yet, the overlying integuments, are not only unbroken, but not even a mark of discoloration may remain, to mark the site of impact.

In no single instance in twenty-five cases of serious abdominal crushes witnessed by myself was there such visible discoloration of the abdominal integument, as in any definite manner, pointed to the probable extent of intramural injury.

Perforation of the bowel or damage to the vascular apparatus is produced by, and succeeds, as a result of the action of, such force as produces various traumatic lesions, elsewhere; and is of two varieties.

First, *when violence immediately* disorganizes, without the action of secondary pathological changes; as when the intestine is immediately ruptured, a vessel is torn open, or an organ lacerated. In this class, the organ is generally crushed against the spinal bodies, which, in the lumbar region are powerful and quite immovable. The action brought into operation is quite the same as we witness in the chopping of wood. We may regard the descending axe as the vertical plummet, and the rachidian buttress as the block.

Now, if the axe falls with reduced force, the stick is merely indented; or if it falls flatwise, it leaves little or no impression.

In ruptures, however force may be appplied, its action must be concentrated and over a very limited area. Possibly tension may play a subsidiary part, but can be little more, for in lacerating the peritoneum the sero-cellular membrane which anchors the intestinal coils to the spinal column, is an elastic and fragile structure, which is easily torn off, on moderate force.

Secondly. *Consecutive pathological changes* are what chiefly lead to serious organic changes in those cases of complicated abdominal injuries. An organ may be so contused and lacerated, that a considerable area of it is devitalized. A cessation of function in the involved part follows, atrophic changes, wasting or resorption of its cellular elements, ulceration, gangrene or suppuration follows. A hollow viscus or large blood-trunk has borne the brunt of injury. We know, from experimentation, that when an intestine or a large artery is severely contused its inner and middle coats give way first. With an artery a coagulum at once forms at the seat of injury and there remains until the processes of repair are complete, and the circulation will suffer probably, but little or any, as it is maintained by the collateral branches; but unhappily for the injured intestine, there are no communicating branches to divert the alimentary current when a local injury is borne by its inclosing walls.

Yet, the economy makes provision for this emergency. Function in the whole intestinal tube is immediately arrested. It is placed at once n a quiescent state, and the remarkable plastic property of the peritoneum is called into prompt action, to seal up the impending breach, through adhesive inflammation.

When a main blood-trunk, like abdominal aorta is damaged, the extremities are threatened.

Denonvillier (*Memoires de chirurgie*, vol. xii, p. 419) has recorded the case of a boy run over by a heavy vehicle.

The abdominal symptoms were severe for a few days, and then showed signs of amelioration, when gangrene appeared on both lower extremities. After death the intima and muscularis were discovered, widely lacerated just above the bifurcation; the lumen was firmly plugged, by a large, partly organized clot. One can understand how the femorals are exposed to crushing force from below, where they pass over the sharp ridges of the pubes; and, the aorta, where it rises from the left to right, to mount the body of the fourth lumbar vertebra.

In spite of the provisions of nature, perforation of the intestine may follow at a late date after, when we feel quite assured that all danger is past. In three cases within the past year, coming under my observation, it suddenly set in just at a time when the patients wanted to get up, and when all severe constitutional disturbances were past; in one on the fourth day ,in another on the eighth day, and another at the end of the third week.

*Traumatic peritonitis* as a consecutive phenomenon to non-penetrating injuries of the abdomen, is caused, chiefly, in three ways.

The first, and most common, is through the direct effects of the injury itself by contusion; in precisely the same manner as trauma of other serous membrane, which line the great cavities and joints. The peritoneum has been bruised or stretched; its nerves have been overstrained or lacerated and there probably has been such an injury to its vascular branches, as leads to congestion or a sanguineous effusion into its subserous stroma of cellular tissue. We may be warned of its advent by the decubitus of the patients, the paretic intestine and hard, sensitive, abdominal surface.

But, inasmuch as the character of the injury which produced it is local, it generally occupies but a limited area, and is often unattended with severe systemic disturbances.

Intra-peritoneal hemorrhage is a prolific source of peritoneal inflammation. When the blood leaves its vessels and escapes into any of the tissues, it becomes an irritant; it is a foreign body and provokes inflammation. This we see well illustrated in a sanguineous extravasation, consecutive to injury at the knee-joint.

We may sometimes, after a severe injury of the abdomen, detect sanguineous fluid in the flanks. Moderate leakage will excite but slight irritation, while a large loss of blood with profound anæmia, can only occur when there is laceration of a solid organ or a large blood-trunk. In the latter class, death may occur before reactionary inflammation of the peritoneum, like that following severe contusion.

*Infective peritonitis* succeeds abdominal contusions, as a rule, when there is a considerable breach in some of the hollow or tubular organs. It is generally conceded, that healthy bile, pancreatic juice, chyle or urine, are not a source of infective or serous inflammation, unless the quantity of escape is very considerable.

The most fatal complication, is when the bowel's wall gives way and the peritoneum becomes infected by intestinal gas or fæces.

In all the cases of direct intestinal rupture which I have examined *post-mortem*, there was a very large associate hemorrhage.

In these cases, death occurred before peritonitis developed. The most dangerous type of traumatic inflammation of the peritoneum comes from consecutive perforation of some part of the intestinal canal. Its advent is sudden and the constitutional symptoms alarming—there is a species of peritoneal shock.

The appendix is sometimes exposed to pressure as it hangs over the pelvic brim, in movements of the infant's head in the parturient act. Probably the healthy caudal appendage, because of its vermicular properties, may easily glide out of the way, as the head engages; but, with a preternaturally long appendix, which has been fixed to the fascia, by previous adhesive inflammation, it is greatly exposed to dangerous compression. Possibly, not a few of those cases set down as salpingitis on the right side, after labor, are nothing other than a severe perforating or non-perforating type of appendicitis.

One such case came under my care this year, succeeding a very difficult labor. From the time of delivery, she complained of a severe pain in the right side; but it was not until the third week, that fulminant symptoms developed. Prompt operation cut them short; when a perforation of the appendix inclosed in a large purulent accumulation was exposed. Another case illustrating the effects of injury as a causative factor in appendicitis, came under my care lately. The patient, a boy of sixteen, was injured by a fall on the right groin.

He took the bed with signs of subacute peritonitis, was up, quite well on the eighth day. At this time he passed from under my care. The next day acute peritonitis set in and he died. On autopsy, there was found a large fæcal extravasation from an opening in the appendix.

*Renal Injury.*—The kidney suffers a trauma through force applied, laterally or posteriorly. It is seldom damaged by vertical force, for the reason, probably, that the intensity of impact is spent, before the organ is reached.

A violent blow over the loin or a fall sustained here, is a prolific source of laceration of the organ, with interstitial bleeding which drains off through the ureter; or we may have an extreme rent with a large accumulation within the capsule. In very severe cases, the capsule itself is torn through, and there is a free escape into the loose, retroperitoneal tissues. This triple complication followed in a case which terminated mortally, under my care early this year. The injured man had fallen from the cabin of a drawbridge, striking on the side over an iron rail twenty feet below. He was a very heavy man and fell with great force. In about all our cases of severe contusion over the lumbar spine we will find evidences of renal complication. Hæmaturia is an early and persistent symptom. It is self-evident that violence over the hypogastrium in the pregnant, must be attended with danger to the fœtus; and, in any one to the bladder, when it is distended. But, the bladder for an organ which seems so much exposed, is very rarely ruptured by force; except, when there is associate fracture of the pelvic bones.

It is not infrequently contused and its inner coats lacerated, when transient hæmaturia occurs, but a complete rupture is a very rare accident indeed. It probably escapes by an alteration of its shape, or by sinking into the pelvis, when distended, making lodgment for itself by pushing the perineum outward. In no other manner can it be explained, how it so generally escapes, while its neighboring organs above are so frequently compromised.

*115 West Forty-ninth Street.*

